# Examining the Intermedia Agenda Setting Effects amid the Changsheng Vaccine Crisis: A Computational Approach

**DOI:** 10.3390/ijerph20054052

**Published:** 2023-02-24

**Authors:** Jian Shi, Hanxiao Wang

**Affiliations:** 1 Academy of Contemporary China and World Studies, China Foreign Languages Publishing Administration, Beijing 100037, China; 2 School of Journalism and Communication, Nanjing Normal University, Nanjing 210097, China

**Keywords:** intermedia agenda setting, network agenda setting, LDA modeling, vaccine

## Abstract

Scholars have long questioned whether the traditional media effects approach can still be applied in the current digital media era, especially in the non-Western, state-regulated Chinese media environment. This study examines the intermedia agenda setting of traditional media sources and we-media sources in the *WeChat Official Accounts* through a computational look at the Changsheng Bio-technology vaccine (CBV) crisis. Utilizing LDA topic modeling and Granger causality analysis, results show that both traditional media and we-media (i.e., online news sources operated by individuals or collectives) focus more consistently on two frames, the *news facts* and the *countermeasure and suggestion* frames. Interestingly, the traditional media agenda impacts the we-media agenda under the *news fact* and the *countermeasure and suggestion* frames, while the we-media agenda influences the traditional media agenda under the *moral judgment* and *causality background* frames. Overall, our study demonstrates the mutual effects between the traditional media agenda and the we-media agenda. This study sheds light on the theoretical meaning of network agenda setting and extends its application to social media platforms in Eastern countries and health-related fields.

## 1. Introduction

Vaccine adverse events have triggered public distrust of proposed immunization measures and may increase the number of populations who are hesitant to participate in these vaccinations. For the past decade, China has seen some cases with the abundance of unregulated vaccines being circulated illegally. For instance, Pang’s Company, a lucrative black-market distributor, has been doing business in illegal vaccines since 2010, having sold 25 varieties of vaccines worth USD 82,000,000 to 24 provinces [1]. In July 2018, according to *The South China Morning*, the Changsheng Bio-technology vaccine (CBV) company was found to have injected more than 200,000 children in Shandong province with batches of faulty vaccines which were untested and included out-of-date materials [1]. In many recent cases, such as those referenced, it was difficult for the public to distinguish the truth from rumors about available vaccines, leading to great concern and fear and, ultimately, a decline in vaccination rates [2,3]. This decline has a huge impact on not only the development of domestic infectious disease prevention but also the normal operation of the entire vaccine industry. Therefore, distributing the right information about vaccines to the public is integral to re-inspiring their trust in disease-prevention measures and protocols. Subsequently, the media platforms that circulate this information are vital to preventative measures with which countries such as China and the United States ultimately move forward.

At present, research on Chinese news coverage of public health security incidents concerning vaccines and infectious diseases is focused mainly on the analysis of the news coverage framework and on the crisis management of public opinion. Using social media data, scholars developed different machine learning models to predict public opinion frameworks [4]. In recent years, WeChat, the Chinese equivalent of Facebook, has gradually become the main Chinese access point for daily news. As WeChat is an important information source for public access to vaccine safety information, the current study examines the similarities and differences between the way that traditional and we-media sources report on vaccine safety incidents in the WeChat Official Accounts.

The purpose of the current study is twofold. Firstly, for technical reasons, research on the topic of vaccine safety using either traditional media or we-media sources in the WeChat Official Accounts is insufficient. Our study considers the CBV crisis in China as an example and examines Granger causality relationships between traditional media and we-media accounts. To this end, the current study thus filled in a gap and examined the application of the NAS model in a state-regulated media environment in a non-Western country such as China. Secondly, in light of the increasing number of media platforms and their complexity, scholars have long questioned whether a traditional media effects approach, such as agenda setting, can still be applied in the current media environment. Given that the content of traditional media accounts and we-media accounts may largely interact with one another, the current study applies intermedia agenda setting theory to examine whether mutual effects between these two media sources exist. Methodologically, considering a large number of WeChat public accounts instead of utilizing traditional content analysis methodology, this study adopts the LDA model to implement topic extraction and classification.

### 1.1. Network Agenda Setting (NAS) and Intermedia Agenda Setting (IAS)

McCombs and Shaw demonstrated that the first level of agenda setting is that the salience of the media’s agenda could transfer to the public agenda [5]. The second level of agenda setting focused on the attribute-level salience and determined how people think of the issues and objects [6]. In addition to the traditional first- and second-level agenda setting and influenced by the development of cognitive psychology, Guo and McCombs proposed a third level of agenda-setting effects: Network Agenda Setting (NAS) [7]. Borrowing the term “the pictures in our heads”, Guo explains the differences among the three different levels of agenda setting as the following: the first level focuses on “what are the pictures about”, the second level focuses on “the characteristics of these pictures”, and the third level answers the question “what are the pictures in our heads” [8] (p. 54). Unlike the assumption of traditional agenda setting, the third level of agenda setting effects presents an individual’s cognition structure as a network-like pattern. In light of the increasing number of media platforms in the new media environment, the interaction between different news media is drawing scholars’ attention.

Intermedia agenda setting refers to an interaction between different news media agendas [9]. Given the development of the Internet and the presence of new media, scholars have shifted their foci when testing intermedia agenda-setting effects to comparisons of traditional and new media. For example, utilizing elite left-leaning and right-leaning political blogs and newspapers, Meraz demonstrated that traditional media agendas failed to set political blogs’ agendas [10]. However, political blogs were still able to influence the traditional media’s agenda.

Intermedia agenda-setting effects have also been tested in the media environments of Chinese society. Comparing the official websites of traditional media with commercial media websites in China, Jiang and Deng found that traditional media still had a strong impact on setting the agenda for commercial media in China. This impact is likely due to commercial media’s lack of interview rights [11]. Jiang and Deng also proposed a two-level flow model of the salience of topics in which the salience of topics transfers from the official mainstream news media to online media and then flows to the netizen [11].

An analysis of framing can be a complement to agenda setting research by addressing how the way a topic is framed can affect the salience of that topic [12]. For example, by highlighting the intermedia frame setting, Wang and Guo’s analysis of online news and Twitter showed that the direction of the intermedia frame setting changed across time [13]. Welbers defined granularity as an important aspect in the selection of agenda-items, which is neglected in intermedia agenda-setting studies [14]. Given that levels of granularity matter to intermedia agenda setting studies, in our study, we decided to further classify various topics to each frame we identified.

### 1.2. The CBV Safety Issues

Framing has the potential to affect how the public perceives and evaluates the issues it confronts [15,16]. *Media framing* suggests “a central organizing idea or storyline that provides meaning to an unfolding strip of events” [17] (p. 143). In order to examine the projections of the safety issues regarding vaccines in WeChat public accounts in the Chinese environment, framing serves as a theoretical foundation to explain how media representation has reflected and maintained the safety issues regarding vaccines. In other words, scholars utilize framing theory as a method to study the underlying meanings of a given content rather than its manifest meaning [18].

In their study of news coverage of the H1N1 influenza, Huang and Dong identified the current issues as facts, official responses, knowledge-based information, and privacy issues [19]. In light of the news coverage of vaccine safety issues on the WeChat public accounts, Dong and Ban concluded that information noise drowns out the scientific and authoritative opinions offered by traditional media sources, causing confusion among we-media publics [20].

In the case of the CBV safety issues, traditional media circulated significant content on real-time progress and follow-up measures, including problem vaccine batches and compensation funds. Essentially, traditional media assumed the responsibility for reporting vaccine safety issues to the public. Contrastingly, we-media netizens reported primarily on the accounts of involved officials and their families, focusing these reports on the life experiences of these officials and the luxurious lifestyles of them and their families instead of the progress of vaccine batches [21]. Taking into consideration the characteristics of the relevant news reports and combining them with the conclusions from previous research (See Table 1), we propose the following research questions:

RQ1: What topical issues relating to the CBV vaccine safety issue are being discussed by traditional media on WeChat?

RQ2: What topical issues relating to the CBV vaccine safety issue are being discussed by official accounts on WeChat?

Since its emergence, we-media has had a voice to share with traditional media (See Table 1). Some of the organizations relevant to the vaccine-safety issues and information about the people directly involved were further exposed by netizens via “real name searching”. The event was first exposed in we-media then covered and its facts disseminated by traditional media. In the meantime, according to the two-step flow of the communication model introduced above, traditional media, in the framework of *news facts* and *countermeasures*, may still have an ability to set the agenda for we-media. Accordingly, we propose the following hypotheses:

**Hypothesis** **1** **(H1).** *In the case of the Changsheng Bio-technology vaccine (CBV) safety issues, Wechat traditional media accounts are more likely to influence we-media accounts under the framework of news facts and countermeasures.*

**Hypothesis** **2** **(H2).** *In the case of the Changsheng Bio-technology vaccine (CBV) safety issues, Wechat we-media accounts are more likely to set an agenda for traditional media accounts under the framework of moral judgment and causality background.*

## 2. Methodology

**Figure 1:**
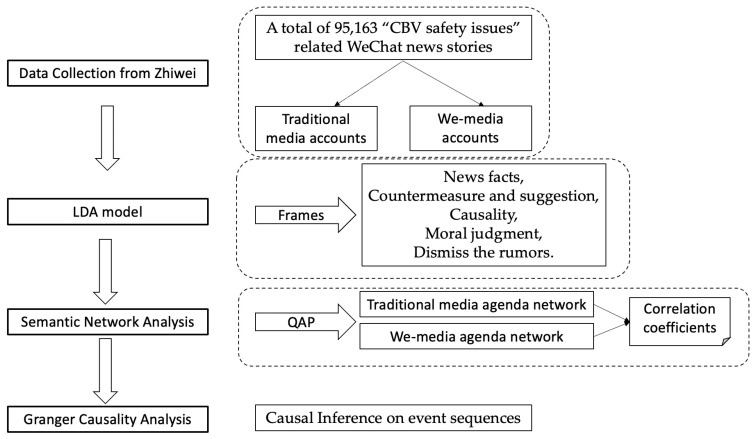
Methodology Flowchart.

As a first step after collecting a comprehensive dataset consisting of news articles covering the (CBV) safety issues, we sorted the news coverage into categories by topic and then renamed each category as a separate frame. We used the Latent Dirichlet Allocation (LDA) approach to determine the probability distribution of each distinct topic in each news story. Next, we assessed intermedia agenda setting (IAS) between traditional media sources and we-media sources on the WeChat Official Accounts. Specifically, we explored the cooccurrence relationships among topics, analyzing the agenda network of various media platforms in WeChat. Finally, a Granger causality model was used in order to facilitate a time-series analysis that classified topics into two distinct groups, one comprising situations for which the traditional media content set the agenda of we-media content. The other group comprised situations for which the we-media content set the agenda of traditional media content (reverse agenda setting) (See Figure 1).

### 2.1. Data Collection and Classification

Utilizing Zhiwei, a data collection company specializing in social media data collection in China [22,23,24], the current study tracked a total of 95,163 “CBV safety issues” related WeChat news stories from 15 July 2018 to 10 August 2018. After collecting the data, our next task was distinguishing and classifying different types of WeChat public accounts because there is no official classification provided by WeChat. We divided the name of each account into separate words and counted each word’s usage frequency. We found a set of specific key words for distinguishing traditional media accounts from organizational media accounts. We looked for 26 feature words, including “daily newspaper” and “morning post” to identify traditional media accounts, and we looked for 35 feature words such as “center” and “agency” to identify organizational media accounts. Utilizing *Regular Expressions* to match feature words, we classified accounts into traditional media and organizational media. Other public accounts that belonged neither to traditional media nor to organizational media were identified as we-media accounts. To ensure the reliability of this classification approach, we randomly sampled 1000 articles in a pretest. The accuracy rate remained 0.72, which was acceptable. In our complete dataset, there were 3185 articles published by traditional media accounts, 13,960 by organizational accounts, and 78,018 by we-media accounts.

### 2.2. LDA Model

As a second step, we utilized LDA to analyze topics among all WeChat public accounts as the total of 95,163 WeChat titles was too large for manual coding. LDA is an unsupervised machine learning tool that utilizes a relational approach to identify document collections and capture connotative meaning and even topic information [25]. Compared to traditional manual coding, LDA can deal with large amounts of data and better describe each document by its distribution of topics. Importantly, LDA could effectively prevent researchers from presetting categories and could report some unexpected results [26]. Not only can this help us generate hypotheses as well as topics that can be easily interpreted by humans, it is also a tool for providing a good set of structures for predictive models [27]. In order to better distinguish similar topics, we adapted Yu and Ma’s classification methods, classifying topics into five different frames. These frames were namely “news facts”, “countermeasure and suggestion”, “causality”, “moral judgment”, and “dismiss the rumors” [28].

### 2.3. Semantic Network Analysis

This study utilized semantic network analysis, which assumes that words appearing together in the same paragraph or in the same article have an impact on audience awareness. In the current study, the co-occurrence relationships reflect the topic’s probability within the title of a single news story. Each title might contain two or more topics. Next, we utilized the Quadratic Assignment Procedure (QAP) to compare the traditional media agenda network and the we-media agenda network. QAP evaluates the coefficient between one sample’s matrix and another sample’s matrix without making parametric assumptions about the data [29]. We first calculated the matching coefficient between equivalent cells within two matrices and then recalculated the matching coefficient of each different random permutation.

### 2.4. Granger Causality Analysis

Granger causality reflects the sequence of time instead of reflecting the real causality [30]. We determined each day as a suitable *time lag* in the *Granger causality* test [10]. In order to test our hypotheses, we used Granger causality analysis to verify the accuracy of the network agenda setting between news stories from the WeChat traditional accounts and from the WeChat we-media accounts.

## 3. Findings

The title of each story was coded using the LDA approach. Before training the topic model, we needed to choose the best number of topics (*k*) for LDA. Based on coherence and perplexity values, we tested various k values, including 5, 10, 15, 20, 25, and 30. After comparing all the LDA models’ results and content from our samples, the results turned out to be clearest when the k value was 25. We then listed the top ten keywords under each different topic. We found that topic 12 and topic 22 focused on the hog cholera vaccine and HPV; therefore, we excluded these two topics from our analysis.

We classified the other 23 topics into five frames, namely *news facts*, *countermeasure and suggestion*, *causality*, *moral judgment*, and *refute rumors with popular science*. In terms of the CBV crisis, multiple media outlets had different foci of the discussion during different periods. Media identified the current issues as announcing factual information, suggesting remedies, diagnosing causes, making moral judgments, and delivering knowledge orientation information. Overall, the media is inclined to stress news facts and to announce official responses and mention fewer private issues.

### 3.1. News-Facts Frame

The news facts frame comprised the news coverage of the CBV scandal, the development of the scandal case, and the government’s reply and actions toward the scandal (See Table 2). Topics 6, 7, and 24 mainly addressed the updated development of the scandal case, such as “Gao was Arrested”, “The State Food and Drug Administration Launch an Investigation on the CBV Scandal”, and “Where did the Unsafe Vaccine Go?” Topics 3, 11, and 16 focused on the investigation team of the State Council, the State Food and Drug Administration, other government departments, and some organizations that investigated the vaccine safety issues together. Additionally, topic 23 involved news coverage of the stock prices of the CBV company as they fluctuated up and down.

### 3.2. Countermeasures and Suggestions Frame

The countermeasure and suggestion frame included two different foci: (1) the announcement issued by the authoritarian department (Guowuyuan) and (2) suggestions on vaccine injection in Hong Kong and some developed countries (See Table 2). Excluding topic 1, listed in Table 3, all other topics belonged to the first focus of the countermeasure and suggestion frame, which involved the vaccine injection details for children; the information about the CBV; and some countermeasure for those who already took the unsafe vaccine. Due to the increasing vaccine safety problems in China, a large amount of news coverage paid attention to Hong Kong, as well as some developed countries’ safety management rules for vaccines and their countermeasure for all kinds of vaccine incidents. For example, topic 1 focused on vaccine injection procedures and countermeasures in Hong Kong under the titles of “Quick Questions About Vaccine Injection in Hong Kong” and “Hong Kong will be a Safer Choice for Vaccine Injection”.

### 3.3. Causality Frame

As Table 4 shows, the causality frame focused on providing a comprehensive timeline of the major aspects of examining the vaccine incidents as well as the lessons the industry learned from the vaccine safety issues. For example, topic 14 focused on the outcome of the vaccine safety issues under titles such as “Will CBV Incidents Influence the Whole Vaccine Industry?” and “Vaccine Pros and Cons”.

### 3.4. Moral Judgment Frame

The moral judgment frame involved queries about the case and emotional venting (See Table 5). Both topic 8 and topic 20 addressed personal information from the persons who oversaw the production of the CBV vaccine and even their families. Examples include “Privatization: the Cause of Vaccine Fraud and the Birth History of the Vaccine Queen”, and “Gao’s Daughter in Law Plays High-profile on the Internet”. Topic 9 included critiques of the inadequate government regulation and coverage of alleged corruption by some local officials, such as “Vaccine incident: 35 drug regulatory officials from 15 provinces got fired!” Topic 25 included critiques of pseudo-vaccine events by journalists such as Yansong Bai and Yongyuan Cui.

### 3.5. Refute Rumors with Popular Science Frame

As Table 6 shows, there is only one topic that belonged to the refute rumors with popular science frame, including the safety vaccine injection procedure. Examples included news stories under the title of “Video About Dismissing the Vaccine Rumors”, “How to Make Sure the Injected Vaccine is Effective?” and “There’s Some Scientific Knowledge You Need to Know About Vaccines”.

After we calculated the co-occurrence frequency, the constructed topic co-occurrence matrices of traditional media news stories and we-media news stories were imported into a social network analysis tool, Ucinet6, to separately calculate their degree centrality. We measured the degree centrality by determining the degree of connection between one node and others. The higher the degree centrality of a node, the more important the position of that node within its network.

Table 7 showed the degree centrality of both the traditional media agenda network and the we-media agenda network. The degree centrality of topics in traditional media under the news facts, countermeasures and suggestions, moral judgment, causality background, and refute rumors with popular science frames are 6852, 7826, 3168, 1030, and 495, respectively; while the degree centrality of topics in we-media under these frames are 116,022, 148,698, 73,600, 33,325, and 13,437, respectively. We observed that traditional media and we-media news stories focus on the news fact and countermeasures and suggestions frames in their coverage of this event. Interestingly, compared to traditional media, we-media news stories mostly focused on moral judgment among the top seven frames. Therefore, RQ1 and RQ2 were both answered.

Importantly, topic 21 under the frame refute rumors with popular science was in a relatively marginal position in both the traditional media agenda network and the we-media agenda network, failing to be the focus of their coverage.

Based on the QAP analysis, we found that the media agenda network was positively associated with the we-media agenda network (r = 0.609, *p* < 0.01). In order to investigate the causality relationship between the traditional media agenda and the we-media agenda, we were especially interested in identifying the topics where traditional media set the we-media agenda and the topics where we-media set the agenda.

We conducted a Granger causality analysis of 25 topics from both the traditional media agenda and the we-media agenda. We first performed a unit root test for the time series. The results showed that all the topics were stable except topics 9 and 14. We then conducted first-order splitting for topics 9 and 14. Finally, the split items were analyzed by Granger causality analysis and passed the co-integration test.

Table 8 shows the results of Granger causality analysis. Topic 16 (Governments and enterprises at all levels collaborated to rectify fake vaccines) and topic 17 (CDC answered questions about the vaccine event) rejected the null hypothesis “traditional media agenda is not the Granger reason for the we-media agenda”. Topic 6 (relevant responsible person of CBV have been Arrested), topic 14 (organization of the whole process of the vaccine fraud event), and topic 20 (Gao Junfang’ s daughter-in-law lives in luxury) rejected the null hypothesis “we-media agenda is not the Granger reason for traditional media agenda”.

Our findings show that there was mutual agenda setting between traditional media and we-media; therefore, both hypotheses 1 and 2 were supported. Specifically, the traditional media agenda had an impact on the we-media agenda under the “news fact” and “countermeasure and suggestion” frames, while the we-media agenda influenced the traditional media agenda under the moral judgment and causality background frames. An exception was topic 6, which showed that we-media also had an impact on traditional media under the “news fact” frame. We assumed that this might be because we-media can more rapidly promote topics of public concern than traditional media after the release of information about the relevant events by government departments.

## 4. Discussions

### 4.1. Key Findings and Implications

We examined intermedia agenda setting between traditional media account coverage and we-media account coverage regarding the CBV crisis on the WeChat Official Accounts. Consistent with Yu and Ma, we empirically identified five frames amid the CBV crisis, namely news facts, countermeasures and suggestions, causality, moral judgment, and refute rumors with popular science [28]. The results showed that the traditional media agenda is strongly associated with the we-media agenda, especially focusing on the news fact and countermeasure and suggestion frames. We-media account news coverage largely focused on the moral judgment frame but did not consider authoritative coverage of traditional media as information noise. It is worth noting that the total amount of coverage under the refute rumors with popular science frame from both traditional media accounts and we-media accounts was relatively low, which was consistent with Ji et al.’ s findings [31].

We conducted Granger causality analyses of both media account platforms. Consistent with previous studies on IAS, we found that traditional media does not necessarily set the agenda for we-media in the digital media era. Instead, they have interacted with each other on a variety of frames [32]. Interestingly, unlike previous studies, we did not find reciprocal relationships between the two media agendas [33]. The results of the current study showed that traditional media can still influence we-media agenda on the news fact and countermeasure and suggestion frames, while we-media can have an impact on the traditional media agenda under the frames of moral judgment and causality background. Traditional media can set the agenda for we-media by relying on its own resource advantages to release authoritative information. We-media’s combination of events and in-depth exploration of the background of relevant responsible persons may, on the other hand, influence the news coverage direction of traditional media.

For traditional media accounts, we suggest expanding its relevant coverage, including more professional advantages. In order to enhance the public’s knowledge of vaccination, traditional media accounts should explain incidents of vaccine classification, vaccination taboos, precautions, and post-vaccination response measures to the public in an easier way.

### 4.2. Limitations

Several limitations should be considered. Firstly, this study does not include government media accounts in its analysis. However, in some concrete examples we collected, we-media forwarded the government announcement before traditional media. That is one of the reasons why we found that the we-media agenda influenced the traditional media agenda under the news facts frame. Future research should take government accounts into consideration and incorporate the policy agenda with our existing frames. Secondly, similar to previous studies, this study set the time interval for Granger causality analysis to one day. Although setting the time interval as one day is more suitable for analyzing cases with longer durations, such as political campaigns, this time interval led to mutual agenda setting appearing between traditional media and we-media under some relevant frames in the current study. For future studies, it is necessary to further shorten the time interval, because the information diffusion process within social media is extremely rapid, changing in just hours or even minutes. We should also note that the vaccine case we have examined is a faulty vaccine case. The results may need to be carefully generalized to other types of vaccine safety issues. We encouraged scholars to examine various types of vaccine safety issues for future studies, which allowed us to collect more evidence regarding the application of IAS in the health-related domain.

## 5. Conclusions

The rapidly changing mediascape requires potential applications of the traditional media effects theories. Overall, this study provides a novel insight into exploring the theoretical and practical meaning of network agenda setting. We have applied intermedia agenda setting to the most popular news source platform in China, namely WeChat Official Accounts. This study also presents the NAS model in a health-related domain in China, which allowed the analysis of communication effects to be tested in a non-Western country. From a theoretical perspective, the study enriched the application of agenda setting effects, moving from previous news websites and microblogs (Chinese Twitter) to WeChat Official Accounts. Methodologically speaking, we solved, to some extent, the problem of classifying WeChat public accounts. According to account naming rules, we distinguished between traditional media, we-media, and organizational media. In this study, we did not explore organizational media accounts. Secondly, to determine the number of topics from each news story, this study used an unsupervised learning technology LDA topic model rather than a traditional content analysis method and manual coding. Next, we constructed the co-occurrence relationship between multiple topics under the same document through using the probability of topics. Thirdly, unlike previous studies, we assumed that news stories set agendas among media platforms based on different frames rather than based on a single topic. Using the LDA topic model to classify the co-occurrence relationships between topics within each news story, we manually combined topics into frames based on Ji et al.’s framework classification and constructed the probability of each topic coverage [31].

## Figures and Tables

**Table 1:** The potential topical issues and proposed framework.

Media Type	Potential Topical Issues	Proposed Framework	Description
Traditional Media	Real-time progress and follow-up measures	News facts and countermeasures	The news facts frame comprised the news coverage of the CBV scandal, and the development of the scandal case; the countermeasures comprised the suggestions on vaccine injection in Hong Kong and some developed countries.
	Investigations on problem vaccine batches		
	Compensation funds		
We-media	Life experiences of the officials	Moral judgment and causality background	The moral judgment frame involved queries about the case and emotional venting; the causality frame focused on providing a comprehensive timeline of the major aspects of examining the vaccine incidents.
	The luxurious lifestyles of them and their families		
	Causality background		

**Table 2:** News Facts Frame.

Topic	Keywords
Topic 3	the State Council Investigation Team, authoritative statement, antibiotics, clinical treatment, Rabies, company CEOs, arrested, Changsheng Bio-technology Vaccine, Li Keqiang
Topic 6	publicity, citizen, phone call, arrested, the public, investigation, exposure, photo, detention center
Topic 7	announcement, in progress, release, authority, conversion to Bumen, right now, litigation, investigation, fine, sale
Topic 11	rabies, Changsheng Bio-technology Vaccine, the Health Care Committee, violation of regulations, illegal production, the State Food and Drug Administration, vaccination, investigation, significant progress
Topic 16	government, traditional Chinese medicine, matters, costs, Yun Ma, children, poison vaccine, overnight, WeChat circle, vaccine incident
Topic 23	Wuhan Bio-technology, delisting, health, formal, doubt, DPT, truth, qualified, iceberg
Topic 24	expired, tracking, service, Japan, the Health Care Committee, vaccination, the National Food and Drug Administration, specification, rabies, Africa

**Table 3:** Countermeasures and Suggestions Frame.

Topic	Keywords
Topic 1	Hong Kong, provide, physical check-up, questions and answers, Gao Junfang, reservation, 9 -valent vaccine, children, exposure, strategy
Topic 2	explain, vaccination, Shan xi, consultation center, Hospital, designated, special, Shang luo, notification, CDC
Topic 4	Interpretation, vaccine, address, list, most comprehensiv, collections, Hepatitis B Vaccine, medical Staff, R&D, FAQ
Topic 5	replant, Q&A, work, health, announcement, list, vaccination, vaccine, rabies vaccine, voluntary
Topic 10	vaccination, rabies, clinic, notification, department, issued, Changchun Changsheng, rabies vaccine, Guangdong, phone consultation
Topic 13	Health and Family Planning Commission, designated hospital, answer, vaccine event, vaccination, implementation, seven days, remind, parents, Shandong Province
Topic 17	Injection, life, fake vaccine, Q&A, online, children, Tiktok, toxic milk powder, parents, investigation
Topic 18	repalnt, announcement, list, release, vaccination, almighty, save, vaccine, agency, price
Topic 19	plan, prevention, consultation, mass, backstage, bid evaluation, progress, formal, disease, Sichuan

**Table 4:** Causality Frame.

Topic	Keywords
Topic 14	understand, China, judgment, huge, injection, thinking, shocked, vaccine, industry, alarm
Topic 15	diffusion, in trouble, cancer, surface, emergence, pregnancy, imprisonment, commissioner

**Table 5:** Moral Judgment Frame.

Topic	Keywords
Topic 8	Gao Junfang, medical institution, vaccine queen, charge, blockchain, wrecker, disruptive, Son, Zheng Yuanjie, extravagant
Topic 9	official, place, amazing, terrible, supervision, pregnant women, safety hazard, counter-feiter, management, quality
Topic 20	bombshell, daughter-in-law, exposure, prison, fake vaccine, whole city, life, flaunt the wealth, fake, luxury
Topic 25	unite, risk dying, chairman, baby, state-owned enterprises, support, Bai Yansong, motion, Cui Yongyuan, family

**Table 6:** Refute Rumors with Popular Science Frame.

Topic	Keywords
Topic 21	Observe, science, finally, fake medicine, message, behind, mode, vigilance, press conference, replant

**Table 7:** Degree Centrality of Agenda Network.

Traditional Media Topic	Frame	Degree Centrality	We-Media Topic	Frame	Degree Centrality
Topic 11	News Facts	1516	Topic 25	Moral Judgment	21,428
Topic 10	Countermeasures and Suggestions	1330	Topic 6	News Facts	21,339
Topic 6	News Facts	1249	Topic 5	Countermeasures and Suggestions	20,869
Topic 5	Countermeasures and Suggestions	1194	Topic 11	News Facts	20,493
Topic 2	Countermeasures and Suggestions	1022	Topic 1	Countermeasures and Suggestions	19,443
Topic 24	News Facts	968	Topic 20	Moral Judgment	18,505
Topic 7	News Facts	964	Topic 9	Moral Judgment	18,387
Topic 3	News Facts	950	Topic 19	Countermeasures and Suggestions	17,886
Topic 1	Countermeasures and Suggestions	905	Topic 2	Countermeasures and Suggestions	16,958
Topic 23	News Facts	849	Topic 10	Countermeasures and Suggestions	16,864
Topic 8	Moral Judgment	839	Topic 15	Causality	16,819
Topic 25	Moral Judgment	805	Topic 14	Causality	16,506
Topic 13	Countermeasure and Suggestion	770	Topic 7	News Facts	16,496
Topic 20	Moral Judgment	763	Topic 24	News Facts	15,527
Topic 9	Moral Judgment	761	Topic 8	Moral Judgment	15,280
Topic 18	Countermeasures and Suggestions	720	Topic 23	News Facts	15,022
Topic 19	Countermeasures and Suggestions	716	Topic 4	Countermeasures and Suggestions	14,939
Topic 4	Countermeasures and Suggestions	663	Topic 13	Countermeasures and Suggestions	14,810
Topic 15	Causality	585	Topic 3	News Facts	14,144
Topic 17	Countermeasures and Suggestions	506	Topic 18	Countermeasures and Suggestions	13,535
Topic 21	Refute Rumors with Popular Science	495	Topic 21	Refute Rumors with Popular Science	13,437
Topic 14	Causality	445	Topic 17	Countermeasures and Suggestions	13,394
Topic 16	News Facts	356	Topic 16	News Facts	13,001

**Table 8:** Granger Causality Analysis.

Topic	Traditional Media Agenda Is Not the Granger Reason for the We-Media Agenda	We-Media Agenda Is Not the Granger Reason for Traditional Media Agenda
news fact		
Topic 3	0.018	0.177
Topic 6	0.004	5.391 *
Topic 7	0.236	0.013
Topic 11	0.578	0.25
Topic 23	0.07	2.573
Topic 16	7.157 **	1.677
Topic 24	0.138	1.458
countermeasures and suggestions		
Topic 1	0.415	0.214
Topic 2	0.144	0.59
Topic 4	0.211	0.19
Topic 5	0.465	0.542
Topic 10	0.04	0.877
Topic 13	0.176	0.358
Topic 17	60.023 **	0.477
Topic 18	0.413	0.758
Topic 19	0.304	0.31
causality background		
Topic 14	0.074	30.608 **
Topic 15	0.059	2.374
moral judgment		
Topic 8	0.346	1.736
Topic 9	0.309	1.704
Topic 20	0.197	3.583 *
Topic 25	0.64	0.633
refute rumors with popular science		
Topic 21	0.64	0.633

Note. * *p* < 0.05, ** *p* < 0.01.

## Data Availability

The data presented in this study are available on request from the corresponding author.
